# Investigating the brain’s neurochemical profile at midlife in relation to dementia risk factors

**DOI:** 10.1093/braincomms/fcae138

**Published:** 2024-04-17

**Authors:** Maria-Eleni Dounavi, Elizabeth McKiernan, Michael Langsen, Sarah Gregory, Graciela Muniz-Terrera, Maria Angeles Prats-Sedano, Marius Ovidiu Mada, Guy B Williams, Brian Lawlor, Lorina Naci, Clare Mackay, Ivan Koychev, Paresh Malhotra, Karen Ritchie, Craig W Ritchie, Li Su, Adam D Waldman, John T O’ Brien

**Affiliations:** Department of Psychiatry, School of Clinical Medicine, University of Cambridge, Cambridge, CB2 0SP, UK; Department of Psychiatry, School of Clinical Medicine, University of Cambridge, Cambridge, CB2 0SP, UK; Center for Clinical Brain Sciences, University of Edinburgh, Edinburgh, EH16 4SB, UK; Centre for Dementia Prevention, University of Edinburgh, Edinburgh, EH16 4UX, UK; Centre for Dementia Prevention, University of Edinburgh, Edinburgh, EH16 4UX, UK; Heritage College of Osteopathic Medicine, Ohio University, Athens, OH 45701, USA; Department of Psychiatry, School of Clinical Medicine, University of Cambridge, Cambridge, CB2 0SP, UK; Medical Research Council Cognition and Brain Sciences Unit, University of Cambridge, Cambridge, CB2 7EF, UK; Department of Clinical Neurosciences and Wolfson Brain Imaging Centre, University of Cambridge, Cambridge, CB2 0QQ, UK; Institute of Neuroscience, Trinity College Dublin, University of Dublin, Dublin, D02 PX31, Ireland; Institute of Neuroscience, Trinity College Dublin, University of Dublin, Dublin, D02 PX31, Ireland; Department of Psychiatry, Oxford University, Oxford, OX3 7JX, UK; Department of Psychiatry, Oxford University, Oxford, OX3 7JX, UK; Department of Brain Sciences, Imperial College Healthcare NHS Trust, London, W12 0NN, UK; INM, Univ Montpellier, INSERM, Montpellier, 34090, France; Centre for Dementia Prevention, University of Edinburgh, Edinburgh, EH16 4UX, UK; Department of Psychiatry, School of Clinical Medicine, University of Cambridge, Cambridge, CB2 0SP, UK; Sheffield Institute of Translational Neuroscience, University of Sheffield, Sheffield, S10 2HQ, UK; Center for Clinical Brain Sciences, University of Edinburgh, Edinburgh, EH16 4SB, UK; Department of Brain Sciences, Imperial College Healthcare NHS Trust, London, W12 0NN, UK; Department of Psychiatry, School of Clinical Medicine, University of Cambridge, Cambridge, CB2 0SP, UK

**Keywords:** MRS, APOE4, Alzheimer’s disease, preclinical dementia, brain metabolism

## Abstract

Changes in the brain’s physiology in Alzheimer’s disease are thought to occur early in the disease’s trajectory. In this study our aim was to investigate the brain’s neurochemical profile in a midlife cohort in relation to risk factors for future dementia using single voxel proton magnetic resonance spectroscopy. Participants in the multi-site PREVENT-Dementia study (age range 40–59 year old) underwent 3T magnetic resonance spectroscopy with the spectroscopy voxel placed in the posterior cingulate/precuneus region. Using LCModel, we quantified the absolute concentrations of myo-inositol, total N-acetylaspartate, total creatine, choline, glutathione and glutamate-glutamine for 406 participants (mean age 51.1; 65.3% female). Underlying partial volume effects were accounted for by applying a correction for the presence of cerebrospinal fluid in the magnetic resonance spectroscopy voxel. We investigated how metabolite concentrations related to apolipoprotein ɛ4 genotype, dementia family history, a risk score (Cardiovascular Risk Factors, Aging and Incidence of Dementia -CAIDE) for future dementia including non-modifiable and potentially-modifiable factors and dietary patterns (adherence to Mediterranean diet). Dementia family history was associated with decreased total N-acetylaspartate and no differences were found between apolipoprotein ɛ4 carriers and non-carriers. A higher Cardiovascular Risk Factors, Aging, and Incidence of Dementia score related to higher myo-inositol, choline, total creatine and glutamate-glutamine, an effect which was mainly driven by older age and a higher body mass index. Greater adherence to the Mediterranean diet was associated with lower choline, myo-inositol and total creatine; these effects did not survive correction for multiple comparisons. The observed associations suggest that at midlife the brain demonstrates subtle neurochemical changes in relation to both inherited and potentially modifiable risk factors for future dementia.

## Teaser

Dounavi and McKiernan et al. used Magnetic Resonance Spectroscopy in a large mid-life cohort with varying future dementia risk. Lower N-acetylaspartate associated with parental dementia; lifestyle risk correlated with higher myo-inositol, choline, creatine and glutamate+glutamine. Neurochemical changes were found at mid-life in relation to inherited and potentially modifiable dementia risk.

## Graphical Abstract

**Graphical abstract:**
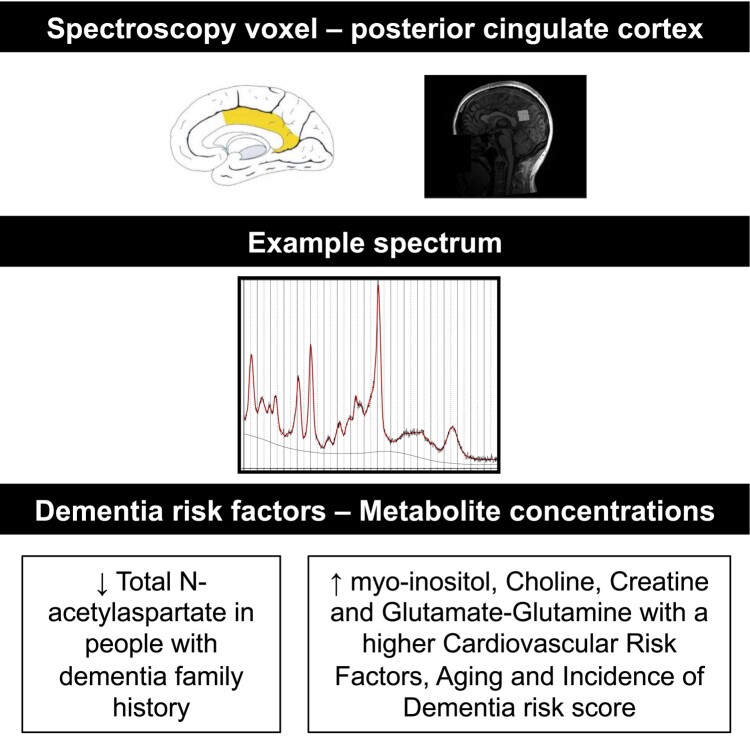

## Introduction

It is increasingly evident that changes in brain physiology and function in Alzheimer’s disease (AD) occur years or even decades before detectable brain structural changes, which themselves precede cognitive deficits.^1,2^ The neurochemical profile has also been reported to change early, during the preclinical stage of the disease.^3^ Magnetic resonance spectroscopy (MRS) allows for the non-invasive measurement of brain metabolite concentrations by exploiting the differences in their resonance frequencies. By acquiring an MRS spectrum in a specified brain volume, it is possible to evaluate the local neurochemical profile. Using MRI field strengths of 3 tesla (3T), several metabolites can be accurately measured including myo-Inositol (mI), N-acetylaspartate (NAA), choline (Cho), creatine (Cr) and glutamate-glutamine (Glx). Metabolite alterations have been found in AD and mild cognitive impairment (MCI) compared with normal ageing; most consistently lower NAA and higher mI.^4-6^ Alterations have also been found in other dementias including dementia with Lewy bodies (DLB), Parkinson’s disease dementia (PDD) and frontotemporal dementia (FTD) compared to normal ageing. Spatial patterns of metabolite changes are different between different dementia types, for example, changes in AD are reported largely in the posterior cingulate cortex (PCC) and hippocampus while in FTD frontal changes predominate^7^. A summary of metabolites detectable at 3T and relevant to studies of AD can be found in Table 1.

**Table 1:** Quantified metabolites of interest with LCModel together with their physiological role and findings in the AD literature

Metabolite	Physiological Role	Findings in the AD continuum
N-acetylaspartate + N-acetylaspartylglutamate (tNAA)	Marker of neuronal density and viability, involved in the regulation of osmosis, myelin lipid synthesis, and mitochondrial energy metabolism.	↓ in AD compared to MCI ↓ in MCI compared to controls
myo-Inositol (mI)	Marker of microglial activation (suggesting an inflammatory process), oedema and amyloid deposition.	↑ in AD compared to MCI ↑ in MCI compared controls
Choline compounds (Cho)	Primarily reflects levels of glycerophosphocholine (GPC) and phosphocholine (PHC). Marker of cell membrane turnover.	Findings not consistent; reports of both ↓ and ↑
Phosphocreatine + Creatine (tCr)	Involved in energy storage and metabolism	Scarcity of reports; may be ↓ in AD and MCI
Glutathione (GSH)	Marker of oxidative stress.	Findings not consistent, tends to be ↓ in AD.
Glutamate + Glutamine (Glx)	Glutamate is the main excitatory neurotransmitter in the human brain. Glutamine is a precursor for glutamate and GABA (main inhibitory neurotransmitter).	Findings not consistent, tends to be ↓ in AD.

In MRS studies focused on AD, voxels are commonly placed in the PCC and/or precuneus, as this is one of the earliest areas affected by AD pathology. Furthermore, voxels placed in this region have: relatively little cerebrospinal fluid (CSF), high grey matter content and improved spectral quality compared with other possible target areas (e.g. hippocampus, which lies close to tissue interfaces that adversely affect local magnetic field homogeneity).^8^ Typically, metabolite concentrations have been investigated as ratios (metabolite/Cr or NAA/mI). However, in recent years and following the publication of expert consensus criteria^9^ a water-reference volume is now more commonly collected which allows for the reporting of ‘absolute’ metabolite concentrations. In the PCC, mI and mI/Cr have consistently been found to be higher in AD compared to MCI and higher in MCI than controls, while NAA and NAA/Cr have been found to be lower in MCI and AD and to decrease as dementia progresses.^5,6^ Myo-inositol is thought to be a marker of gliosis and may reflect neuroinflammation, while NAA reflects neuronal density.^10^

Alterations have also been reported in the concentration of other metabolites, though less consistently. Creatine may be lower in MCI and AD^7^ but has been found to increase with age.^11^ Both increases and decreases in Cho in AD have been reported, with the lack of consistency potentially attributable to the routine usage of acetylcholinesterase inhibitors.^7^ Concentrations of Glx and GSH (which are more difficult to measure at lower MRI field strengths) have been found to be lower in AD^5^. Mixed findings in recent reviews and meta-analyses reflect the heterogeneity of MRS studies which may be attributable to several factors such as small participant numbers, variable voxel placement, reporting of ratios or ‘absolute values’ and analysis choices.

Previous MRS studies of cognitively normal adults at high risk of future dementia have examined carriers of familial mutations such as presenilin (PSEN) 1 and amyloid precursor protein (APP—related to early onset AD) reporting lower NAA and higher mI and Cho in presymptomatic and mildly symptomatic mutation carriers in the PCC/precuneus.^12,13^ Studies investigating changes in preclinical sporadic AD populations have stratified participants based on apolipoprotein ɛ4 (APOE4) carriership (the most common risk gene for sporadic AD^14^), amyloid and tau levels in cerebrospinal fluid (CSF) or using positron emission tomography (PET). In cognitively-normal older adults, studies have found that a higher amyloid load or amyloid positivity was connected with increased mI/Cr, Cho/Cr and mI/NAA in the PCC/prenuceus.^3,15^ Furthermore, it was shown that mI/Cr increases in APOE4 carriers may precede amyloid pathology.^3^ In a longitudinal study investigating metabolite concentration changes it was shown that changes in mI/Cr and NAA/mI were associated with amyloid pathology.^16^ When the relationship between baseline PCC MRS and longitudinal amyloid PET in cognitively-normal older participants (mean age 74 years) was explored, higher mI/Cr and lower NAA/mI at baseline were associated with increased amyloid accumulation, a relationship which was not modified by APOE4 status.^17^ Finally, studies examining the effect of APOE4 on brain metabolites have reported either non-significant effects on the concentrations^18^ or a higher mI/Cr in APOE4 carriers.^3^ Thus, well-established AD biomarkers, but not necessarily APOE4 status, have been shown to correlate with metabolite concentrations in older cognitively normal adults at higher risk of sporadic AD.

Studies investigating changes in relation to dementia risk factors have so far focussed on older participants (mean ages in the eighth decade of life) and on markers of heritable risk such as APOE4 status or established AD biomarkers (e.g. positive amyloid PET) rather than potentially modifiable risk factors such as those related to cardiovascular risk. In terms of dietary patterns, links between adherence to a Mediteranean diet and decreased dementia risk have been demonstrated^19^ and MRS metabolite concentrations have been found to alter in response to altered dietary intake.^20^ A recent systematic review of MRS at MRI field strengths of 3T or above found only one study of ‘at risk’ individuals at midlife and no papers which systematically examined the relative effects of potentially modifiable and non-modifiable dementia risk factors on MRS metabolite concentrations.^7^

In the present study our aim was to investigate the concentrations of brain metabolites in the PCC/precuneus region in a midlife cohort in relation to risk factors for future AD. We examined metabolite concentrations in relation to both non-modifiable and potentially modifiable dementia risk factors in the PREVENT-Dementia cohort. Risk stratification was based on APOE4 genotype, dementia family history (FHD), the Cardiovascular Risk Factors, Aging, and Incidence of Dementia (CAIDE)^21^ risk score and its constituents (sex, age, years of education, cholesterol, exercise, body mass index and blood pressure) and dietary patterns. Based on previous studies investigating metabolite concentrations in the disease’s continuum, we hypothesized that we would observe higher mI and lower NAA in participants at higher risk of developing AD (APOE4+, FHD+ or with potentially modifiable risk factors).

## Materials and methods

### The cohort

Participants in the PREVENT-Dementia study were aged between 40 and 59 years and were cognitively normal at the time of recruitment as determined during a thorough clinical examination. The recruitment target was 50% with and 50% without parental dementia family history.^22,23^ The study was approved by the London-Camberwell St Giles National Health Service Ethics Committee (REC reference: 12/LO/1023) which operates according to the Helsinki Declaration of 1975 (and as revised in 1983) and by the Trinity College Dublin School of Psychology Research Ethics Committee (SPREC022021–010) and the St James Hospital/Tallaght University Hospital Joint Research Ethics Committee. All participants provided written informed consent. Exclusion criteria for the study were a diagnosis of MCI or dementia and known MRI contraindications. More details on the study population can be found in Ritchie and Ritchie 2012 and Ritchie *et al*. 2013.^22,23^ Taqman genotyping was used to determine the APOE genotype from DNA extracted from blood.

### Imaging protocol

**Figure 1:**
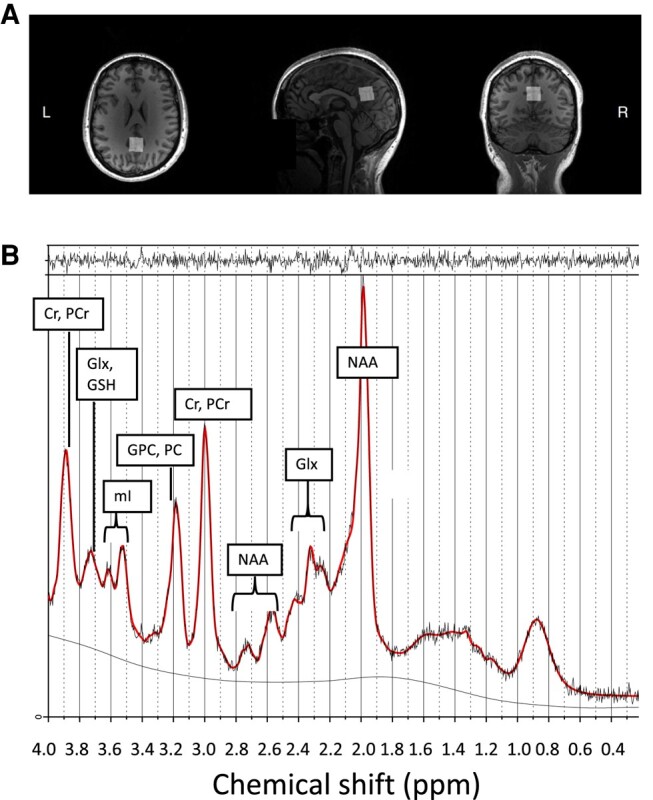
**MRS voxel placement and spectrum.** (**A**) placement of the posterior cingulate cortex/precuneus 20 × 20 × 20 mm^3^ voxel in one participant, (**B**) an example MRS spectrum with metabolites of interest labelled generated with LCModel version 6.3. Cr, creatine; PCr, phosphocreatine; Glx, glutamate + glutamine; GSH, glutathione; mI, myo-Inositol; GPC, glycerophosphocholine; PC, phosphocholine; NAA, N-acetylaspartate (signal also includes N-acetylaspartylglutamate).

MRS data were acquired on 3T Siemens clinical MRI systems (Siemens Medical Solutions, Erlangen, Germany) in four study sites across the UK and Ireland (Prisma: Edinburgh; Prisma fit: Cambridge; Verio: West London, Edinburgh and Skyra fit: Edinburgh, Skyra: Dublin) for 508 participants as part of a multi-modal MRI protocol. For the present study data from magnetization prepared rapid gradient echo (MPRAGE: repetition time—TR = 2.3 s, echo time—TE = 2.98 ms, 160 slices, flip angle = 9^°^, voxel size = 1 mm^3^ isotropic) and single voxel Point RESolved spectroscopy (PRESS: TR = 2 s, TE = 30 or 33 ms, flip angle = 90^o^, voxel size = 20 × 20 × 20 mm^3^, 96 averages) acquisitions were used. An additional PRESS acquisition in the same voxel without water suppression (16 averages) was collected. The spectroscopy voxel was placed in the PCC/precuneus area since it is a region demonstrating progressive degeneration in the course of AD and reproducible voxel placement is easier compared, for example, to the hippocampal region.^8^ Instructions were given to every study site for the consistent placement of the MRS voxel. An example voxel placement is shown in Fig. 1A.

### Metabolite concentration and correction for tissue concentration

LCModel version 6.3 was used for metabolite quantification.^24,25^ The acquisition without water suppression was used for bias field correction and quantification of metabolite concentrations. Metabolites of interest were: mI, N-acetylaspartate & N-acetylaspartylglutamate (NAA + NAAG, tNAA for the rest of the manuscript), Glx, glutathione (GSH), Cho (glycerophosphocholine—GPC and phosphocholine -PCh) and phosphocreatine & creatine (Cr & PCr, tCr for the rest of the manuscript). Two basis sets were generated using simulations optimized for TE, for each TE used in the study (30 and 33 ms). Spectral simulations were performed for 25 common brain metabolites using vendor-specific pulse waveforms and acquisition parameters using the open-source MATLAB-based package FID-A^26^ and with the free, online MRSCloud tool for reduced simulation runtime.^27,28^ Basis sets were generated from these simulations with MRSCloud and LCModel *makebasis* functions. To account for macromolecules and lipids we used the standard LCModel approach which fits independent basis spectra and a flexible spline baseline. The baseline accounts for incompletely supressed water, lipids and macromolecules.^24^

Following quantification of metabolite concentrations, individual spectra were retained if the following three criteria were met: (i) good fit of the spectrum based on visual assessment, (ii) relatively smooth baseline and (iii) all metabolites of interest had a relative Cramér Rao Lower Bound (CRLB) < 30%. An example of a spectrum with good fit is shown in Fig. 1B.

To account for partial volume effects due to CSF, we used Gannet’s stand-alone co-registration script (version 3.1.5) in Matlab (Matlab 2019a, The MathWorks Inc., Natick, MA, USA) between the MPRAGE and MRS acquisition voxel, which relies on SPM routines to perform tissue segmentation.^29^ The fraction of grey matter (GM), white matter (WM) and cerebrospinal fluid (CSF) within every voxel was quantified. CSF contains only minimal quantities of metabolites of interest, mainly glucose, lactate and glutamine.^30^ Hence, analysis results were corrected for presence of CSF in the voxel (partial volume correction, PVC) by multiplying the concentrations of the quantified metabolites with 1/(1-CSF).^31^

### Potentially modifiable risk factors for future dementia

The CAIDE dementia score (version without APOE4) was generated based on information about sex, age, years of education, cholesterol, exercise, body mass index and blood pressure (range 0–15).^21^ These factors were also investigated independently in relation to the quantified metabolites. Furthermore, to evaluate the effect of dietary patterns in the brain’s neurochemical profile, the Mediterranean Diet Adherence Score (MEDAS)^32^ and Medietrranean diet Pyramid^33^ scores were calculated based on detailed food frequency questionnaires and published methodologies. A higher score in each denotes closer adherence to a Mediterranean-style diet which has been associated with reduced risk of dementia.^19^

### Statistical analysis

Statistical analysis was conducted in Matlab 2021b (R2021b, The MathWorks Inc., Natick, MA, USA). The Wilcoxon rank sum test and the *χ*^2^ test were used for between-group comparisons of demographic factors. To account for different scanning sites and TEs, we harmonised the metabolite concentrations using COMBAT, adding as modulating variables: age, sex, years of education, APOE4 and FHD.^34^ COMBAT is a popular technique for harmonization of multi-site neuroimaging data that has recently been validated and used to harmonize MRS data.^35^

The association of each of the outcome variables of interest and a core set of variables including age, sex, years of education, APOE4 and FHD was investigated using robust linear regression analyses. As a first level of analysis, we explored differences between APOE4 carriers and non-carriers and FHD+/FHD− in separate models. The CAIDE score was examined in relation to metabolite concentrations using Spearman correlations. Individual constituents of the CAIDE score (age, sex, education years, SBP, cholesterol, BMI, activity) were examined in relation to metabolite concentrations using robust linear regression models (all factors considered together). To examine the relationship of individual metabolites to diet, robust linear regression models were run with age, sex, years of education and diet scores as predictors.

Results from the linear regression models were corrected for multiple comparisons using the false discovery rate (FDR) method.^36^ Individuals with missing data (demographic/lifestyle variables) were excluded from the relevant analyses.

The following additional exploratory analyses were run: (i) analysis based on three groups for APOE4 genotype (non-carriers, heterozygotes, homozygotes) to examine potential gene-dose effects, (ii) analysis examining maternal and paternal dementia family history separately, (iii) analysis by excluding participants with diabetes since the condition is known to impact the concentration of several metabolites and could be driving the observed associations,^37^ (iv) investigation of APOE4/FHD * age, APOE4/FHD * sex interaction terms, (v) analysis exploring ratios to Cr instead of ‘absolute’ metabolite concentrations corrected for PVC of CSF and (vi) addition of the GM % in the voxel in the linear regression models.

## Results

Overall, 406 (of the 471) participants had MRS spectra of good quality and were free from incidental findings (399 participants for the GSH analysis). The mean SNR was 38.3 ± 9.8 and mean FWHM was 0.062 ± 0.015 (values per study site are shown in Supplementary Table 1; significant site-differences were recorded for both SNR and FWHM). Participants positive for FHD had more WM (*P* = 0.05) and less GM (*P* < 0.01) in the MRS voxel, there was no difference for the APOE4 carrier/non-carrier analysis. The CSF content within the voxels was similar between the examined groups. The mean CAIDE score for the cohort was 4.83 ± 2.53 (15 people were missing data necessary for the calculation of CAIDE). Demographic characteristics for the cohort can be found in Table 2. Quality features for each of the considered metabolites are reported in Supplementary Table 2. Quality control markers of SNR and FWHM were not different between FHD+ and FHD- participants or between APOE4 carriers and non-carriers. There were, however, significant associations between CAIDE and SNR (*ρ* = −0.18, *P* < 0.01) and between CAIDE and FWHM (*ρ* = 0.20, *P* < 0.01). The correlation between different metabolites is shown in Supplementary Fig. 1.

**Table 2:** Demographic specifications for the analysable cohort (shown as percentage or mean ± standard deviation)

	APOE4− (261)	APOE4+ (145)	*P*-value	FHD- (196)	FHD + (210)	*P*-value
Age (years)	51.4 ± 5.4	50.6 ± 5.4	0.10	50.5 ± 5.9	51.8 ± 4.9	**0.05**
Sex (% female)	66.7%	62.8%	0.44	62.8%	67.6%	0.34
Education (years)	16.6 ± 3.4	16.6 ± 3.2	0.79	16.7 ± 3.6	16.5 ± 3.1	0.43
BMI (kg/m^2^)	27.4 ± 5.7	27.6 ± 5.0	0.40	27.4 ± 5.0	27.6 ± 5.9	0.86
Cholesterol (mmol/L)	5.5 ± 0.9	5.6 ± 1.0	0.21	5.5 ± 1.0	5.6 ± 1.0	0.45
SBP (mmHg)	124.5 ± 16.6	124.5 ± 14.4	0.78	124.0 ± 16.7	124.9 ± 15.0	0.35
MEDAS	5.6 ± 1.7	5.6 ± 1.8	0.95	5.6 ± 1.7	5.6 ± 1.8	0.79
Pyramid	8.2 ± 1.5	8.2 ± 1.6	0.55	8.2 ± 1.5	8.2 ± 1.6	0.97
GM (%)	67.0 ± 4.8	66.2 ± 4.6	0.10	67.6 ± 4.4	66.0 ± 5.0	**<0.01**
WM (%)	20.3 ± 4.7	21.0 ± 5.2	0.24	20.0 ± 4.3	21.1 ± 5.4	**0.05**
CSF (%)	12.8 ± 4.7	12.8 ± 4.5	0.94	12.6 ± 4.5	13.0 ± 4.7	0.53

Missing values for 39 participants for MEDAS score and 32 for Pyramid. Bold is used to indicate significant differences at a level of *P* = 0.05. APOE4, apolipoprotein ɛ4; BMI, body mass index; FHD, family history of dementia; GM, gray matter SBP, systolic blood pressure; WM, white matter.

### Dementia risk and metabolite concentrations

**Figure 2:**
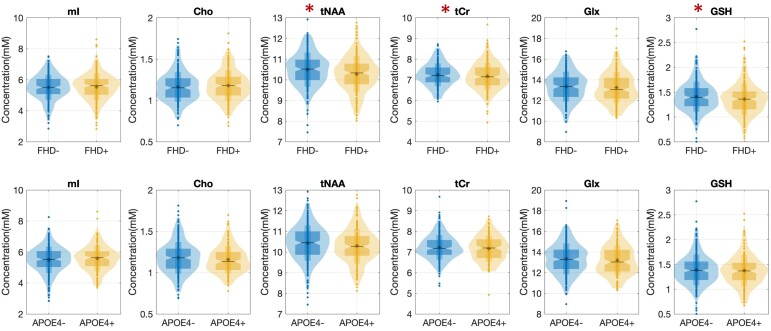
**Metabolite concentration differences between people with and without dementia family history and between carriers and non-carriers of the APOE4 gene.** An asterisk in front of a metabolite indicates significant group differences. The Matlab al_goodplot extension was used for the plot generation. The asterisk within the plots corresponds to the mean, median is shown as a bold line, the boxplot spans from the 1st to the 3rd quartile. The overlapping violin plot spanned from the 1st to the 99th percentile and the outline matches the kernel density. Statistical test results (*t* statistic from linear regression models): mI (*t*_FHD_ = −0.13, *p*_FHD_ = 0.89; *t*_APOE4_ = 0.55; *p*_APOE4_ = 0.58), Cho (*t*_FHD_ = 0.85, *p*_FHD_ = 0.40; *t*_APOE4_ = −1.35; *p*_APOE4_ = 0.18), tNAA(*t*_FHD_ = −3.06, *p*_FHD_ <0.01; *t*_APOE4_ = −1.74; *p*_APOE4_ = 0.08), tCr (*t*_FHD_ = −2.00, *p*_FHD_ = 0.05; *t*_APOE4_ = −0.39; *p*_APOE4_ = 0.70), Glx (*t*_FHD_ = −1.24, *p*_FHD_ = 0.22; *t*_APOE4_ = −1.36; *p*_APOE4_ = 0.18), GSH (*t*_FHD_ = −2.02, *p*_FHD_ = 0.04; *t*_APOE4_ = −0.79; *p*_APOE4_ = 0.43). APOE4, apolipoprotein ɛ4; Cho, choline; FHD, dementia family history; Glx, glutamine-glutamate; GSH, glutathione; mI, myo-inositol; mM, millimolar; tCr, total creatine; tNAA, total N-acetylaspartate.

Detailed results for group differences between FHD+/− and between APOE4+/− are shown in Fig. 2. Participants with FHD had lower tNAA (*t* = −3.06, *P* < 0.01, *p*_FDR_ = 0.01), tCr (*t* = − 2.00, *P* = 0.05, *p*_FDR_ = 0.09) and GSH (*t* = −2.10, *P* = 0.04, *p*_FDR_ = 0.09). Since the GM and WM content of the MRS voxel differed between people with and without dementia family history we further included GM in the linear regression model. Following inclusion of this term FHD remained significantly associated only with tNAA (*t* = −2.75, *P* = 0.01).

**Figure 3:**
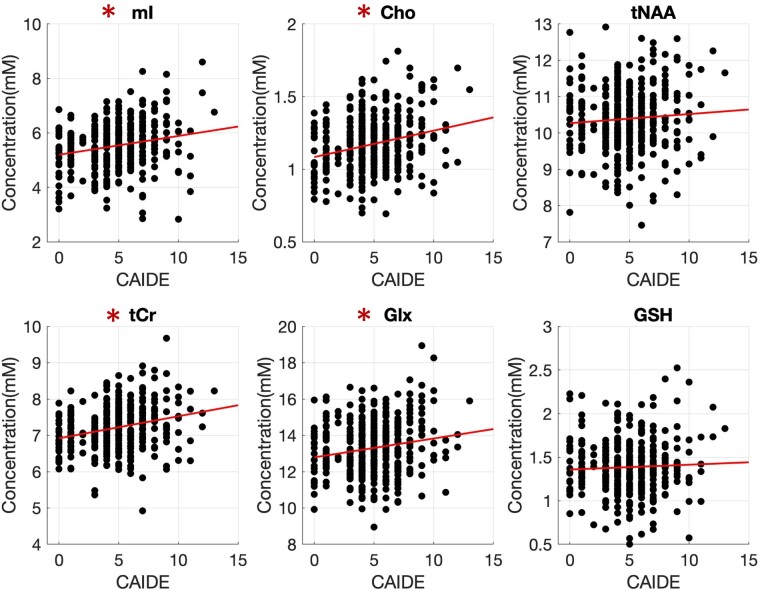
**Relation of metabolite concentration to the CAIDE score.** Relation of metabolite concentration to the CAIDE score for 391 participants (384 for GSH). The fitted line corresponds to first order polynomial fitting. Asterisks are used to indicate a significant association with CAIDE following Spearman correlations; mI—CAIDE (*ρ* = 0.17, *P* < 0.01), Cho (*ρ* = 0.21, *P* < 0.01), tNAA (*ρ* = 0.08, *P* = 0.14), tCr (*ρ* = 0.26, *P* < 0.01), Glx (*ρ* = 0.16, *P* < 0.01) and GSH (*ρ* = 0.08, *P* = 0.11). CAIDE, Cardiovascular Risk Factors, Aging and Incidence of Dementia; Cho, choline; Glx—glutamine-glutamate; GSH, glutathione; mI, myo-inositol; mM, millimolar; tCr, total creatine; tNAA, total N-acetylaspartate.

In Fig. 3 associations between metabolite concentration and the CAIDE score are shown. Associations were observed between CAIDE and mI (*ρ* = 0.17, *P* < 0.01, *p*_FDR_ < 0.01), Cho (*ρ* = 0.21, *P* < 0.01, *p*_FDR_ < 0.01), tCr (*ρ* = 0.26, *P* < 0.01, *p*_FDR_ < 0.01) and Glx (*ρ* = 0.16, *P* < 0.01, *p*_FDR_ < 0.01). A higher CAIDE score was associated with higher CSF in the voxel (*ρ* = 0.27; *P* < 0.01) and lower GM (*ρ* = −0.19; *P* < 0.01). To investigate if the observed effects could be attributed to lower GM present in the voxel with a higher CAIDE we further examined linear regression models with CAIDE and GM as predictors. All observed associations remained significant, with a further positive association with tNAA (*t* = 2.17; *P* = 0.03, *p*_FDR_ = 0.04).

### Associations with potentially modifiable risk factors and diet

**Figure 4:**
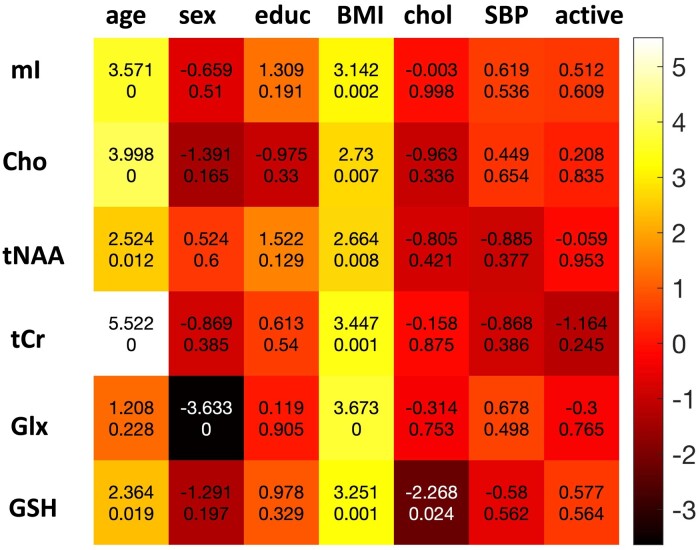
**Association of dementia risk factors with metabolite concentrations.** The intensity of the boxes reflects the t-statistic from linear regression models, values within the boxes are shown as t-statistic at the top and *P*-value at the bottom for the corresponding predictors in the multiple linear regression models. A *P*-value of ‘0’ indicates the *P* was <0.001. BMI, body-mass index; chol, cholesterol; Cho, choline; educ, education; Glx, glutamine-glutamate; GSH, glutathione; mI, myo-inositol; SBP, systolic blood pressure; tCr, total creatine; tNAA, total N-acetylaspartate.

In Fig. 4 the results of robust linear regression models with age, sex, years of education, BMI, cholesterol, SBP and activity as predictors are shown. Higher age and BMI were positively associated with the majority of quantified metabolites. Greater adherence to a Meditteranean diet as indicated by a higher MEDAS score was related to significantly lower Cho (*t* = −2.26; *P* = 0.02, *p*_FDR_ = 0.15). Associations were also recorded between greater adherence to a Meditteranean diet as indicated by a higher Pyramid score and lower mI (*t* = −2.03, *P* = 0.04, *p*_FDR_ = 0.15) and tCr (*t* = −1.97, *P* = 0.05, *p*_FDR_ = 0.15). A total of 39 participants were missing data on the MEDAS score and 32 on the Pyramid score.

### Exploratory analyses

Further exploratory analyses were conducted to investigate the effects of the number of APOE4 copies, paternal and maternal dementia family history and age and sex interactions with APOE4 and FHD in the metabolite concentrations. Additional analysis was run by excluding participants with type-2 diabetes. There were no differences between APOE4 non-carriers and homozygotes (*n* = 26) and between APOE4 non-carriers and heterozygotes (*n* = 119). Sixty-eight participants had paternal FHD, 117 had maternal and 23 participants had both parents diagnosed with dementia. An effect for tNAA was seen for all groups (paternal—*t* = −1.96, *P* = 0.05; maternal—t = −2.29, *P* = 0.02; both—*t* = −3.46, *P* < 0.01). A significant effect was seen in participants with both parents diagnosed with dementia for tCr (*t* = −3.13; *P* < 0.01) and Glx (*t* = −2.30; *P* = 0.02). For GSH there was no significant difference recorded, only trends most strongly for maternal FHD (*t* = −1.85; *P* = 0.06). No significant interaction effects were found between APOE/FHD and sex or age in predicting metabolite concentrations.

Eight participants had a diagnosis of diabetes and were on diabetes medications. When these participants were excluded from the analysis the FHD and APOE4 differences described above and associations with CAIDE remained unchanged. In terms of associations with the constituents of the CAIDE score, the majority of observed effects remained unchanged with the exception of the GSH—cholesterol association which was not significant anymore (*t* = −1.92; *P* = 0.06). The MEDAS score was no longer associated with Cho (*t* = 1.92, *P* = 0.06) and a similar pattern was observed for the association between the Pyramid score and mI (*t* = 1.90, *P* = 0.06) and Pyramid and tCr (*t* = −1.74, *P* = 0.08).

When ratios to Cr were examined, we did not find any differences between APOE4 carriers and non-carriers or FHD+ and FHD−. In terms of associations with CAIDE score, there was only one association between CAIDE and tNAA/Cr (*ρ* = −0.22, *P* < 0.01).

## Discussion

In the present study investigating neurochemical changes in a midlife cohort in relation to risk factors for future dementia, we found relationships between metabolite concentrations in the PCC/precuneus region and both inherited and potentially modifiable dementia risk factors. High risk of dementia due to family history of dementia was associated with lower tNAA. In an exploratory analysis this effect was larger in individuals with maternal rather than paternal risk and larger still when both parents were affected; it has been suggested that maternal family history may have a greater effect on risk than paternal family history^38^ and that risk increases as the number of affected relatives increases.^39^ Lower tCr and GSH in those with a family history of dementia did not persist when additional correction was made for the concentration of GM in the voxel which differed significantly between groups. It needs to be noted that structural differences in this cohort have thoroughly been investigated and there are no group differences in cortical thickness or GM between APOE4+/− and FHD+/−.^40^ There were no differences in metabolite concentrations between APOE4 carriers and non-carriers. A higher CAIDE score incorporating age, sex and potentially modifiable dementia risk factors was associated with higher concentrations of mI, Cho, tCr and Glx. This effect was mainly driven by higher BMI and older age. Our findings suggest that subtle neurochemical changes in the PCC in those with dementia risk factors have potentially started in midlife, and that the metabolites involved and direction of change depends on the type of risk, i.e. inherited versus potentially modifiable.

We found lower tNAA in people with FHD but not in APOE4 carriers. Differences in tNAA concentration between high and low risk groups based on FHD are in line with previous studies investigating metabolite changes early in the dementia trajectory. In a recent meta-analysis of 66 MRS studies investigating MCI and AD, Song *et al*.^5^ found that NAA was consistently lower across brain regions including the PCC in MCI and AD compared to controls. Lower NAA/mI is also reported in MCI and early AD^6^ and it has been shown that baseline values may predict conversion to MCI and dementia.^41^ As a marker of neuronal integrity and mitochondrial energy metabolism,^42^ early alterations in its concentration could potentially relate to neuronal health and metabolism.

Contrary to our hypothesis, we found no associations between inherited dementia risk and mI concentration, although a positive association (in line with our hypothesis) was recorded with CAIDE. Posited to be the metabolite whose concentration changes earliest in the dementia trajectory, increased mI has commonly been interpreted to reflect early neuroinflammatory processes.^43^ Elevated mI has been reported in pre-clinical and ‘at risk’ populations: increased mI was found to be present in a cohort with preclinical familial AD more than 12 years before expected dementia onset^12^; higher amyloid has also been found to be associated with increased mI/Cr in cognitively-normal adults.^3,15^ The association between mI and CAIDE in our cohort was principally driven by increasing age and increasing BMI, with both age and adiposity established as pro-inflammatory states.^44,45^ There is however debate as to the specificity of mI as a marker of glial activation or density^43^ and raised mI in the context of neurodegenerative disease may also reflect secondary osmolyte or second messenger effects related to oedema or amyloid deposition.^46^ Higher adherence to Mediterrenean diet (Pyramid score) was found to relate to lower mI, an association which did not remain significant following FDR or after exclusion of participants on diabetes medication. The lack of associations between heritable dementia risk and mI in our study may be due to the relative youth of our cohort, which has a mean age of 51 years. Previous cognitively healthy cohort studies have focused on older participants, rather than those in midlife. Finally, technical challenges to accurate measurement of mI concentrations from the complex j-coupled resonance are reflected in the higher standard deviation of calculated mI concentrations compared with most other metabolites included, which effectively limits sensitivity to biological change.

Increased Cho was associated with CAIDE, driven by age and BMI; Cho has previously been found to be associated with ageing across multiple brain areas,^11^ while a relationship between Cho and BMI is less well established. Lower Cho was associated with higher MEDAS (a score describing closer adherence to a Mediterranean diet), however this association did not persist when participants with diabetes were excluded in an exploratory analysis, suggesting that it may be driven by lifestyle or genetic factors which are also associated with diabetes. We did not find any difference in Cho concentrations between FHD and APOE groups. Cho concentration reflects cell-membrane turnover; in the MCI and AD literature Cho has been inconsistently reported as both increasing or decreasing, depending on the age of participants, degree of dementia, brain region sampled and methods used.^5,6^

Lower tCr was found in those with FHD+ (an effect which in further analysis was shown to be driven largely by the difference in the voxel’s GM content) while a higher CAIDE score was associated with higher tCr which was again driven by age and BMI. The correlation between CAIDE and tCr may reflect changes occurring secondary to increased vascular risk, such as changes in local vascularization.^46^ Higher Cr has been reported with ageing^11^ and may reflect glial proliferation^18^ but it has also been reported to demonstrate a downward trend in MCI and AD.^5^ Historically, metabolites have more commonly been presented as ratios to Cr rather than as ‘absolute’ concentrations, in part due to issues around measurement reproducibility and avoiding the need to correct for voxel content. Adherence to Mediterrenean diet (Pyramid score) was negatively associated with tCr, an effect which did not persist following exclusion of participants with diabetes or FDR. When we conducted an exploratory analysis using ratios to Cr instead of absolute values corrected for CSF, the only recorded association was a negative association between tNAA/Cr and CAIDE. This may suggest that the negative NAA/Cr association with CAIDE is driven by increasing Cr and highlights that methodological choices can significantly impact the study observations. Our findings support the current understanding that Cr is not an optimal reference metabolite.

We did not find any group difference in Glx between FHD and APOE groups. Glx was found to be positively associated with CAIDE and BMI and to be lower in females. There is some evidence in the literature of lower Glx in AD and MCI.^6,47^ At 3T, without the use of specialised acquisitions that allow spectral editing, it is difficult to resolve glutamate (Glu) from glutamine (Gln) which combined are referred to as Glx. Glutamate is the principal excitatory neurotransmitter in the brain while Gln is a precursor to other metabolites including Glu but also GABA (the main inhibitory neurotransmitter in the brain). The physiological significance of a change in Glx is therefore difficult to ascertain.

Glutathione (GSH) was positively associated with age and BMI and negatively associated with cholesterol, though not with the overall CAIDE score. It was also lower in the FHD group, an effect mainly driven by the difference in GM content in the voxel. GSH is considered a marker of oxidative stress, due to its role removing reactive oxygen species in the brain.^46^ A limited number of studies have looked for changes in GSH in MCI and AD based on the hypothesis that oxidative stress may contribute to the pathogenesis of AD.^5^ Decreases in PCC GSH have been reported in MCI and AD and have been found to correlate with measures of poorer cognition.^48-50^ Furthermore, they have been found to accurately differentiate MCI and AD from controls.^48,49^ The scarcity of studies reporting on GSH may be due to a lack of MRS acquisition optimization. Glutathione is one of the more challenging metabolites to measure accurately as it has several broad low-amplitude signals and cannot be fully differentiated from other metabolites without the use of a specialized GSH acquisition such as using J-difference editing.^48,51^ These challenges are somewhat reflected in our cohort in which the CRLB values for GSH were higher than for the other metabolites, resulting in a higher number of rejected results at the quality control stage, and relatively high CRLB values for those data included.

Given the difference in tNAA, tCr and GSH between FHD groups, the lack of group difference in metabolite concentrations based on APOE4 status may be considered surprising; it is not however incongruent with the current literature, in which the relationship between APOE4 and metabolite concentrations is not straightforward. For example, a study^17^ exploring the relationship between baseline PCC MRS in 594 cognitively-normal older participants (mean age 74 years) and longitudinal amyloid PET found that higher mI/Cr and lower NAA/mI at baseline were associated with increased amyloid accumulation but that this relationship was not modified by APOE4 status, although APOE4 was associated with faster Αβ accumulation. A study of cognitively-normal older adults reported that APOE4 genotype was associated with increased mI/Cr in amyloid negative participants,^3^ while another study^18^ did not find a significant APOE4 effect or APOE4 × age interactions in any metabolites. We did not find any differences between APOE4 non-carriers and homozygotes or between APOE4 non-carriers and heterozygotes in our additional exploratory analyses. Furthermore, we did not find any significant interaction effects between APOE and sex or age. Combined with our findings, these results suggest that the relationship between APOE4/FHD and neurochemistry in cognitively normal adults is complex and likely modified by other factors such as age and amyloid status.

Excluding those participants with diabetes did not impact the differences found between FHD and APOE4 groups. The subgroup with diabetes patients constituted only eight participants who were slightly older (mean age 53) and had higher BMI (mean 35.19) than the overall cohort. Following their exclusion, significant differences in metabolite concentrations in association with the MEDAS and Pyramid diet scores were no longer seen. The associations between MEDAS score and Cho and between Pyramid score and mI and tCr may, therefore be driven by age, BMI or diabetes itself. Further subgroup analysis is limited by the lack of individuals with diabetes in the cohort.

Strengths of the study include the large, well-characterized midlife cohort, thorough quality control, quantification of ‘absolute’ metabolite concentrations, state-of-the-art harmonization and PVC. Differences in MRS acquisition, quality control and PVC techniques, alongside small, underpowered studies, have led to significant heterogeneity in the existing MRS AD, MCI and ‘at-risk of dementia’ literature; several of these limitations have been addressed in this study. The use of ratios to Cr and a lack of PVC in the literature may be particularly concerning in studies considering populations with or at risk of dementia since Cr concentration increases with age and may also be altered in disease as described above. Meanwhile, atrophy is a key marker of disease progression, hence accounting for the tissue composition of the MRS voxel is crucial to ensure that potential detected differences can be attributed to pathology and not to underlying atrophy. Limitations of this study include that the voxel positioning was conducted by different radiographers depending on the site and variations in TE. Our study included only one MRS voxel, placed in the PCC and the amyloid status of our participants was unknown hence we had to rely on APOE4, FHD and CAIDE for risk stratification. The multi-site nature of the study can also be considered a limitation due to the different quality characteristics between different scanning sites which was addressed by using COMBAT harmonization. We applied a post-processing method correcting only for CSF partial volume effects; in the future a PVC method also accounting for GM and WM concentrations prior to fitting with LCModel could be considered. Finally, the MRS sequence utilized limited the number of metabolites we could examine.

Overall, in this large cohort of cognitively normal adults in midlife we found subtle alterations in the brain’s neurochemical profile in participants at increased risk of future AD in relation to inherited (FHD—lower tNAA) or potentially modifiable risk factors (CAIDE; BMI—higher mI, Cho, tCr, Glx). Together these findings imply that both non-modifiable and potentially modifiable risk factors might have a differential impact on the brain’s neurochemical profile at midlife. Further longitudinal studies and investigation of associations with well-established disease biomarkers will be able to shed further light onto the mechanisms leading to the observed changes.

## Supplementary Material

fcae138_Supplementary_Data

## Data Availability

The data that support the findings of this study are available from the corresponding author upon reasonable request.
