# Supplementary material for: Investigating the brain’s neurochemical profile at midlife in relation to dementia risk factors
Source: Brain Commun. 2024 Apr 17;6(3):fcae138. doi: 10.1093/braincomms/fcae138 (PMC11109818; doi:10.1093/braincomms/fcae138)
Supplement: fcae138_Supplementary_Data [file fcae138_supplementary_data.pdf]

Tables

Study Site	SNR	FWHM
West London	38.1 ± 7.6	0.07 ± 0.01
Edinburgh	33.9 ± 9.0	0.06 ± 0.02
Cambridge	45.9 ± 11.5	0.05 ± 0.02
Dublin	40.4 ± 8.4	0.05 ± 0.01

Supplementary Table 1: Signal to noise ratio (SNR) and full width at half maximum (FWHM) for each PREVENT-Dementia study site for the 406 datasets included in the study.

Included (n = 406)		Excluded (n = 65)		
**Metabolite**	**%SD**	**Metabolite**	**%SD**	**Scans with SD >30**
**NAA**	1.63 ± 0.52	**NAA**	4.03 ± 3.24	0
**mI**	6.11 ± 4.26	**mI**	27.39 ± 45.44	12
**Cho**	4.71 ± 1.64	**Cho**	13.93 ± 12.80	8
**Cr**	1.82 ± 0.51	**Cr**	5.07 ± 4.01	1
**Glx**	4.34 ± 1.20	**Glx**	13.83 ± 31.54	1
**GSH**	11.88 ± 49.67<br>**8.73± 3.05 ***	**GSH**	128.07 ± 278.17	22

Supplementary Table 2: Quality features for quantified metabolites for the spectra included in the manuscript as well as the excluded spectra. The values are calculated by excluding values of '999' as returned by LCModel indicative of failed fitting. For the excluded cohort this was the case for the following metabolites: 2 for mI, 1 for Cr, 2 for Cho and 6 for GSH. * The reported values in the second row for GSH are for the subset of 399 participants for which the data have been reharmonised. Abbreviations: Cho – choline; Cr- creatine; Glx – glutamine-glutamate; GSH – glutathione; mI – myo-inositol; NAA –N-acetylaspartate; SD – standard error estimates.

Figures

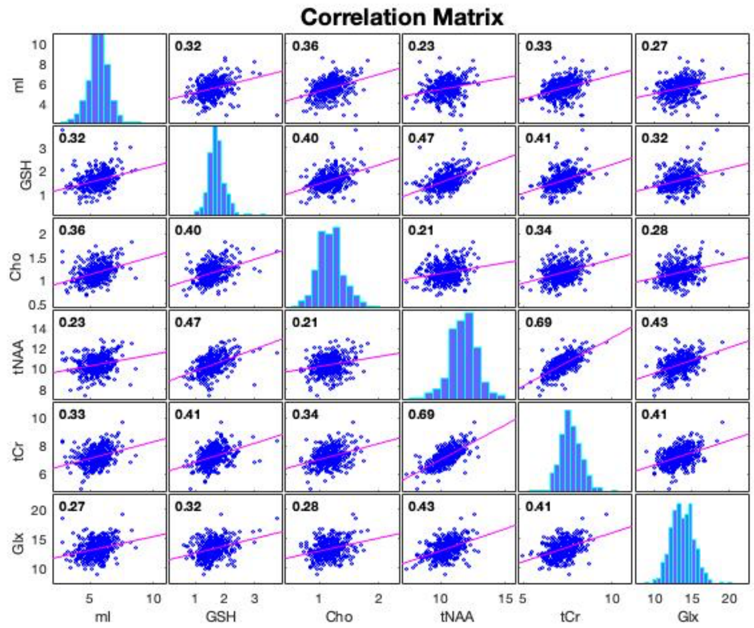

Supplementary Figure 1: Cross-correlation between the considered metabolites. Values shown are the ρ correlation coefficient. Cho – choline; educ- education; Glx – glutamine-glutamate; GSH – glutathione; mI – myo-inositol; tCr- total creatine; tNAA – total N-acetylaspartate
